# Extensive exometabolome analysis reveals extended overflow metabolism in various microorganisms

**DOI:** 10.1186/1475-2859-11-122

**Published:** 2012-09-11

**Authors:** Nicole Paczia, Anke Nilgen, Tobias Lehmann, Jochem Gätgens, Wolfgang Wiechert, Stephan Noack

**Affiliations:** 1 Institute of Bio- and Geosciences, Biotechnology, Systems Biotechnology, Forschungszentrum Jülich GmbH, Jülich, Germany

**Keywords:** Overflow metabolism, Crabtree effect, Exometabolome, Mass spectrometry, Intracellular metabolite quantification

## Abstract

Overflow metabolism is well known for yeast, bacteria and mammalian cells. It typically occurs under glucose excess conditions and is characterized by excretions of by-products such as ethanol, acetate or lactate. This phenomenon, also denoted the short-term Crabtree effect, has been extensively studied over the past few decades, however, its basic regulatory mechanism and functional role in metabolism is still unknown. Here we present a comprehensive quantitative and time-dependent analysis of the exometabolome of *Escherichia coli*, *Corynebacterium glutamicum*, *Bacillus licheniformis*, and *Saccharomyces cerevisiae* during well-controlled bioreactor cultivations. Most surprisingly, in all cases a great diversity of central metabolic intermediates and amino acids is found in the culture medium with extracellular concentrations varying in the micromolar range. Different hypotheses for these observations are formulated and experimentally tested. As a result, the intermediates in the culture medium during batch growth must originate from passive or active transportation due to a new phenomenon termed “extended” overflow metabolism. Moreover, we provide broad evidence that this could be a common feature of all microorganism species when cultivated under conditions of carbon excess and non-inhibited carbon uptake. In turn, this finding has consequences for metabolite balancing and, particularly, for intracellular metabolite quantification and ^13^C-metabolic flux analysis.

## Background

Overflow metabolism, i.e. producing ethanol under aerobic and glucose excess conditions, is one of the outstanding characteristics of a Crabtree-positive yeast such as *Saccharomyces cerevisiae*[1,2]. The Crabtree effect involves glucose-induced inhibition of respiration and oxidative phosphorylation accompanied by a parallel up-regulation of glucose uptake and glycolysis. However, the precise mechanism is still unresolved [3-5]. More recently, it has been discussed that this phenomenon is a general survival strategy of *Saccharomyces* yeast in order to outcompete other microorganisms in fast sugar consumption [6].

In the same way as ethanol in yeast, acetate overflow is well-known for prokaryotic organisms such as *Escherichia coli* and is also termed the bacterial Crabtree effect [7,8]. Acetate overflow predominantly occurs under conditions of high glucose concentrations, i.e. in the exponential growth phase of batch cultures or in chemostat cultures operated at high dilution rates. In contrast, no extracellular acetate accumulation was found under glucose-limiting conditions, i.e. the stationary phase of batch cultures or chemostat cultures operated at low dilution rates [9]. Similar to the Crabtree effect in yeast, its regulatory mechanism in bacteria is still under investigation [10-12].

Interestingly, recent experimental data from shake-flask cultivations of several bacterial species including *E. coli* indicated that metabolites originating from central carbon metabolism are present in the culture media [13]. A similar observation was made for *S. cerevisiae* and *E. coli* in glucose-limited chemostat cultures [14,15]. The fact that this has not been observed before is due to the improved mass spectrometry capabilities allowing concentration measurements down to the nanomolar range. However, except for singular measurements, no systematic investigation on the microorganism’s exometabolome including time-dependent effects of metabolite excretion and uptake has been carried out so far.

In this paper, we address the conundrum related to the presence of metabolites originating from central carbon metabolism in the culture medium during typical bioreactor cultivations. The exometabolomes of four biotechnologically relevant model organisms, i.e. the wild types of *Escherichia coli*, *Bacillus licheniformis* and *Saccharomyces cerevisiae* as well as an L-lysine producing strain of *Corynebacterium glutamicum*, were rigorously and broadly analyzed over the time course of batch cultivations under well-controlled bioreactor conditions. In accordance with these results, several hypotheses to explain the sometimes considerable amounts of metabolites detected in the broth were formulated and tested.

## Results and discussion

### Organism-wide exometabolome analysis

**Figure 1:**
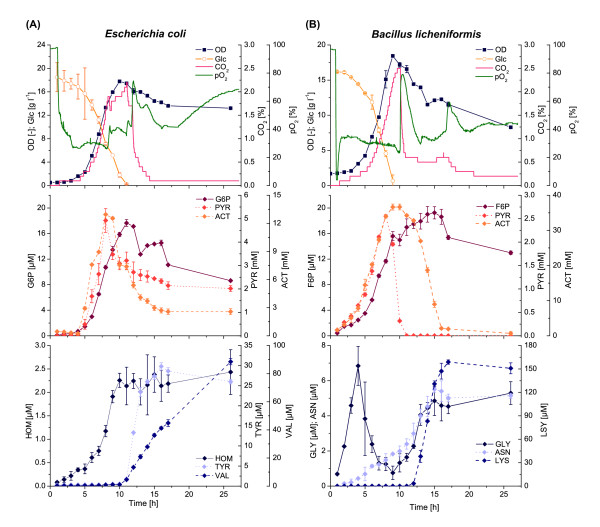
**Exometabolome analysis of *Escherichia coli *and *Bacillus licheniformis*. (A)** Batch cultivation of *E. coli* WT on defined media with 20 g l^-1^ glucose. **(B)** Batch cultivation of *B. licheniformis* WT on defined media with 16 g l^-1^ glucose. Abbreviations: OD, optical density; Glc, glucose; CO_2_, offgas carbon dioxide; pO_2_, dissolved oxygen; G6P, glucose-6-phosphate; PYR, pyruvate; ACT, acetate; HOM, homoserine; TYR, tyrosine; VAL, valine; F6P, fructose-6-phosphate; GLY, glycine; ASN, asparagine; LYS, lysine.

The growth of *E. coli* proceeded in a typical batch mode manner (Figure 1A). During the exponential phase the substrate glucose was converted to biomass and the main by-products carbon dioxide, pyruvate and acetate. Exponential growth stopped about 8 h after inoculation at a remaining glucose concentration of *c*_Glc_ ≈ 6 g l^-1^. In the following transitory phase, glucose was further consumed and the cells also started to take up the preliminary by-products pyruvate and acetate in parallel. When the glucose was nearly exhausted a very short stationary phase was observed, until finally the biomass concentration slowly began to decrease and carbon dioxide formation rapidly dropped.

Unexpectedly, during the whole cultivation process significant amounts of central carbon metabolism intermediates were found in the culture medium (Figure 1A, Table 1 and Additional file 1: Figure S1). Some metabolites were strongly correlated to biomass growth (e.g. glucose-6-phosphate), whereas others showed a time-delayed increase and remained constant after reaching a maximum value (e.g. phosphoenolpyruvate). In contrast, some amino acids such as L-valine were first released by the cells at the beginning of the stationary phase.

**Table 1:** **Extracellular metabolite concentrations in the culture broth of batch cultures of*E. coli*,*B. licheniformis*,*S. cerevisiae*and*C. glutamicum***

**Central metabolic intermediates**	***E. coli*(K12 W3110)**	***B. licheniformis*(DSM13D102)**	***S. cerevisiae*(CEN.PK 113-7D)**	***C. glutamicum*(DM1800)**
Glucose-6-phosphate	17.65 ± 0.53 (t = 11)	88.53 ± 7.64 (t = 26)	1.25 ± 0.01 (t = 26)	10.16 ± 0.74 (t = 182)
Fructose-6-phosphate	29.29 ± 0.60 (t = 10)	19.25 ± 0.96 (t = 15)	0.87 ± 0.01 (t = 17)	3.84 ± 0.22 (t = 182)
Fructose-1,6-bisphosphate	34.21 ± 0.72 (t = 11)	9.41 ± 0.60 (t = 14)	11.43 ± 0.29 (t = 7)	3.36 ± 0.07 (t = 110)
Dihydroxyacetone phosphate	50.40 ± 1.74 (t = 12)	19.75 ± 0.14 (t = 9)	0.41 ± 0.01 (t = 26)	8.67 ± 0.10 (t = 19)
Glyceraldehyde-3-phosphate	15.04 ± 0.24 (t = 12)	2.67 ± 0.05 (t = 8)	< LOD	0.56 ± 0.01 (t = 27)
2/3-phosphoglycerate	19.45 ± 1.55 (t = 16)	66.26 ± 2.01 (t = 15)	2.19 ± 0.01 (t = 26)	12.17 ± 1.07 (t = 182)
Phosphoenolpyruvate	20.43 ± 0.28 (t = 9)	21.76 ± 0.48 (t = 12)	1.08 ± 0.02 (t = 26)	< LOD
Pyruvate	4924 ± 509 (t = 8)	2553 ± 25 (t = 8)	734.5 ± 33.2 (t = 6)	< LOD
6-Phosphogluconate	< LOD	< LOD	< LOD	< LOD
Ribose-5-phosphate	10.29 ± 0.22 (t = 16)	23.03 ± 0.73 (t = 9)	< LOD	0.61 ± 0.03 (t = 0.03)
Ribu-/Xylulose-5-phosphate	3.69 ± 0.04 (t = 8)	12.27 ± 0.38 (t = 11)	0.02 ± 0.01 (t = 17)	0.58 ± 0.05 (t = 182)
Erythrose-4-phosphate	1.31 ± 0.21 (t = 15)	2.04 ± 0.01 (t = 17)	< LOD	2.47 ± 1.14 (t = 18)
Sedoheptulose-7-phosphate	< LOD	< LOD	< LOD	< LOD
Acetyl-CoA	< LOD	< LOD	< LOD	< LOD
Citrate	139.8 ± 6.2 (t = 12)	104.1 ± 3.6 (t = 9)	26.44 ± 0.11 (t = 26)	n.d.
Cis-aconitate	14.13 ± 0.40 (t = 12)	12.27 ± 0.15 (t = 9)	1.65 ± 0.01 (t = 26)	< LOD
Isocitrate	23.13 ± 0.54 (t = 12)	18.28 ± 0.38 (t = 9)	6.12 ± 0.12 (t = 17)	n.d.
α-Ketoglutarate	200.0 ± 17.7 (t = 16)	75.73 ± 1.62 (t = 8)	50.13 ± 0.92 (t = 26)	< LOD
Fumarate	12.45 ± 2.31 (t = 11)	120.8 ± 2.6 (t = 8)	12.35 ± 0.38 (t = 16)	< LOD
Malate	< LOD	< LOD	< LOD	< LOD
Glyoxylate	< LOD	< LOD	< LOD	< LOD
**Cometabolites**				
AXP	< LOD	< LOD	< LOD	< LOD
NAD(P)	< LOD	< LOD	< LOD	< LOD
NAD(P)H	< LOD	< LOD	< LOD	< LOD
**Amino acids**				
Alanine	1.69 ± 0.19 (t = 9)	138.0 ± 2.4 (t = 8)	5.47 ± 1.18 (t = 26)	64.80 ± 4.21 (t = 182)
Leucine/Isoleucine	20.17 ± 0.21 (t = 26)	11.23 ± 0.4 (t = 13)	8.07 ± 0.40 (t = 26)	< LOD
Valine	88.50 ± 2.18 (t = 26)	31.67 ± 1.30 (t = 13)	7.69 ± 0.61 (t = 26)	56.00 ± 6.66 (t = 92)
Aspartate	20.87 ± 0.55 (t = 26)	4.01 ± 0.32 (t = 16)	4.20 ± 0.25 (t = 26)	28.73 ± 0.32 (t = 48)
Homoserine	2.43 ± 0.48 (t = 26)	9.15 ± 0.86 (t = 9)	< LOD	46.61 ± 0.8 (t = 182)
Threonine	2.59 ± 0.30 (t = 14)	< LOD	< LOD	< LOD
Methionine	0.55 ± 0.01 (t = 26)	123.7 ± 3.3 (t = 26)	0.15 ± 0.01 (t = 26)	26.67 ± 0.75 (t = 182)
Lysine	< LOD	158.8 ± 2.8 (t = 17)	3.99 ± 1.52 (t = 9)	5957 ± 80 (t = 182)
Tryptophane	0.25 ± 0.01 (t = 12)	9.15 ± 0.17 (t = 26)	< LOD	< LOD
Tyrosine	29.80 ± 0.90 (t = 16)	67.20 ± 2.80 (t = 26)	1.41 ± 0.08 (t = 26)	8.21 ± 0.36 (t = 182)
Phenylalanine	28.00 ± 0.56 (t = 26)	206.4 ± 4.5 (t = 26)	4.48 ± 0.22 (t = 26)	< LOD
Glutamate	< LOD	55.20 ± 1.39 (t = 8)	28.32 ± 0.56 (t = 26)	180.1 ± 0.9 (t = 17)
Glutamine	< LOD	< LOD	2.71 ± 0.15 (t = 9)	< LOD
Proline	0.54 ± 0.13 (t = 13)	20.71 ± 0.82 (t = 11)	7.40 ± 0.28 (t = 5)	34.69 ± 1.75 (t = 17)
Serine	0.87 ± 0.52 (t = 13)	1.73 ± 0.19 (t = 16)	1.60 ± 0.17 (t = 6)	24.27 ± 1.22 (t = 98)
Glycine	1.23 ± 0.52 (t = 13)	6.84 ± 1.11 (t = 4)	< LOD	698.4 ± 60.2 (t = 182)
Arginine	9.61 ± 0.91 (t = 7)	< LOD	76.80 ± 0.01 (t = 10)	35.47 ± 1.43 (t = 182)
Histidine	6.84 ± 0.01 (t = 8)	58.53 ± 1.40 (t = 26)	10.83 ± 4.54 (t = 10)	< LOD
**Others**				
Acetate	12949 ± 851 (t = 8)	36657 ± 821 (t = 10)	7518 ± 151 (t = 9)	< LOD
Ethanol	n.d.	n.d.	4.58 ± 0.04 (t = 10)	n.d.
Orotate	1100 ± 27 (t = 9)	25.00 ± 4.24 (t = 14)	18.50 ± 0.71 (t = 17)	n.d.
Uracil	1443 ± 30 (t = 26)	2099 ± 45 (t = 26)	175.0 ± 2.8 (t = 26)	n.d.

Each value represents the time dependent maximum during the cultivation. Mean values and standard deviations are estimated from three technical replicates and the time point of occurrence is given in brackets. All concentration values are given in μM. Abbreviations: LOD, limit of detection; n.d., not determined.

The general growth behavior of *B. licheniformis* was comparable to *E. coli* (Figure 1B). Here an exponential growth phase with formation of the same by-products carbon dioxide, pyruvate and acetate was also followed by a short transitory phase. Again the remaining glucose was consumed to continue growth and pyruvate was rapidly taken up. However, acetate was not taken up in parallel, but continued to increase until the beginning of the stationary phase yielding a maximum of 37 mM, which is almost three times higher compared to *E. coli*. At the start of the stationary phase, the carbon dioxide formation immediately dropped, but the cells still actively consumed acetate until it was fully depleted. Similar to the case of *E. coli*, high concentrations of intracellular metabolites were detected in the culture medium (Figure 1B, Table 1 and Additional file 1: Figure S2).

**Figure 2:**
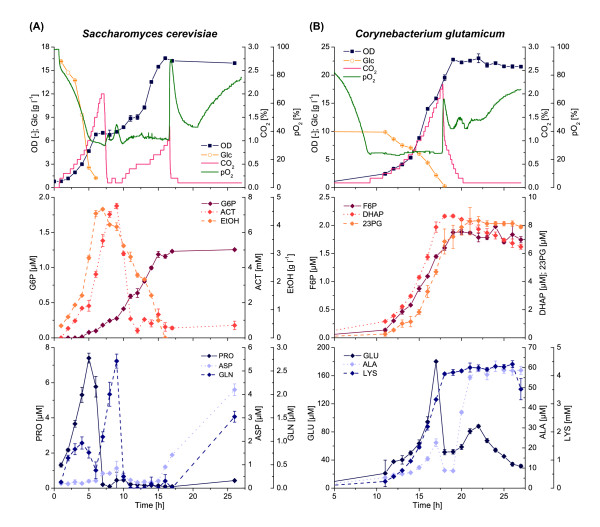
**Exometabolome analysis of *Saccharomyces cerevisiae *and *Corynebacterium glutamicum*. (A)** Batch cultivation of *S. cerevisiae* WT on defined media with 16 g l^-1^ glucose. **(B)** Batch cultivation of *C. glutamicum* DM1800 on defined media with 10 g l^-1^ glucose. Abbreviations: OD, optical density; Glc, glucose; CO_2_, offgas carbon dioxide; pO_2_, dissolved oxygen; G6P, glucose-6-phosphate; ACT, acetate; EtOH, ethanol; PRO, proline; ASP, aspartate; GLN, glutamine; F6P, fructose-6-phosphate; DHAP, dihydroxyacetone phosphate; 23PG, 2/3-phosphoglycerates; GLU, glutamate; ALA, alanine; LYS, lysine.

The batch cultivation profile of *S. cerevisiae* was significantly different showing two distinct growth phases (Figure 2A). During a first short exponential growth phase, glucose was rapidly consumed to form biomass, carbon dioxide, pyruvate (Additional file 1: Figure S3), acetate and to a large extent ethanol. In the subsequent first transitory and stationary phases, growth under glucose limitation and pyruvate uptake was followed by a switch to ethanol and acetate consumption. Thereafter a complete second growth phase on ethanol was initiated, which finally led to a maximum biomass density of OD_600_ = 16, which was comparable to the cultivation experiments with *E. coli* and *B. licheniformis* (both OD_600_ = 18). As already shown for these experiments, time-dependent concentration profiles of nearly all measurable intracellular metabolites were observed in the extracellular medium (Figure 2A, Table 1 and Additional file 1: Figure S3).

The results shown above were derived on the basis of the wild types of *E. coli*, *B. subtilis* and *S. cerevisiae*. In order to extend the scope and relevance of our investigation, we also analyzed the L-lysine producer *C. glutamicum* DM1800 with regard to its exometabolome (Figure 2B, Table 1 and Additional file 1: Figure S4). Although growth of this strain followed a typical batch mode manner as shown for *E. coli* and *B. licheniformis*, no significant by-product formation of pyruvate or acetate was observed. Nevertheless, also in that case metabolites from glycolysis, pentose-phosphate pathway, TCA cycle as well as free amino acids accumulated in the culture medium.

The finding that a broad spectrum of metabolites was detected in the culture medium of all four organisms consequently asks for a reason of their presence. In the following we formulate and evaluate several hypotheses based on results mainly derived from *E. coli* and *C. glutamicum*.

### Cell lysis during cultivation

The most common explanation for the presence of intracellular metabolites in the culture medium is that they stem from cell lysis and, accordingly, the detected metabolite pools are only artifacts. To examine the impact of cell lysis on the exometabolome, we first followed a simple estimation by coupling our *E. coli* data with already published intracellular data of central metabolic intermediates (Additional file 1: Table S1). As an example:

1) Assuming a complete lysis of all biomass formed during exponential growth in our 1 liter bioreactor,i.e. CDW_max_ = 7.8 g (cf. Figure 1A, CDW = 0.44*OD for exponential growth).

2) Taking the maximum literature value for the intracellular concentration of glyceraldehyde-3-phosphate (GA3P) into consideration, i.e.*c*_GA3P,intra_ = 0.52 μmol g_CDW_^-1^[16,17].

The resulting extracellular concentration is calculated as *c*_GA3P,extra_ = 4.06 μM. In comparing this theoretical value with our maximum experimental value of *c*_GA3P,extra_ = 15.04 μM (cf. Table 1), it becomes clear that the GA3P found in the extracellular medium during cultivation can by no means be explained by cell lysis alone. The two values can only be brought into agreement if the biomass is formed more than three times higher as observed during the exponential phase, while at the same time most of this biomass is completely lysed.

Clearly, depending on the reported intracellular value for a certain metabolite (e.g. fructose-1,6-bisphosphate) the amount of lysed biomass necessary to explain the extracellular data could be much smaller (e.g. 10% of CDW_max_, cf. Additional file 1: Table S1). However, this amount is still very high to serve as a reasonable explanation in case of central metabolic intermediates.

For the measured free intracellular amino acids the situation is different because amino acids can also be derived from protease digestion of proteins. Especially, in the case of L-valine where accumulation was first observed at the beginning of the stationary phase (cf. Figure 1A), cell lysis seems reasonable. As another example:

1) Following a reduction in biomass from the exponential to the late stationary phase of ΔCDW = 0.5 g.

2) Assuming a maximum protein content of around 60% in *E. coli*[18] and an L-valine content of 7.4% of total cell protein [19].

3) The molecular weight of L-valine is M = 117 g mol^-1^. However, during protein formation the amino acids are polymerized resulting in loss of water molecules, and hence, the use of M = 99 g mol^-1^ serves here for a better approximation.

As a result, only protease digestion of cellular protein would amount to about 224 uM and this value could reasonable explain the observed extracellular concentration of c_VAL,extra_ = 88.5 μM. Noteworthy, the OD and cell dry weight measurements are not anymore correlated in the stationary growth phase possibly pointing to a change in the cell’s morphology, which in turn might also be a source of amino acids stemming from hydrolysis of membrane proteins without necessarily assuming lysis of these cells (data not shown).

**Figure 3:**
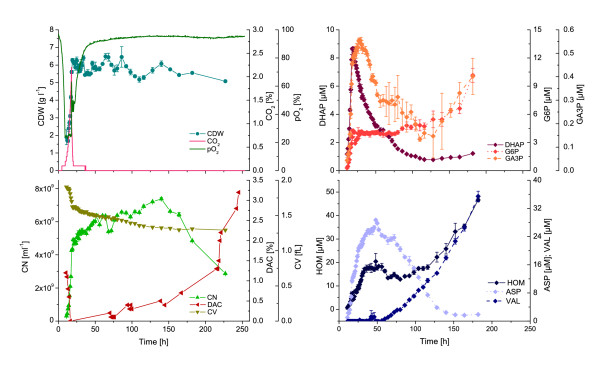
**Impact of cell lysis on the exometabolome of *C. glutamicum *DM1800 during long-term batch cultivations on defined media with 10 g l**^**-1**^**glucose.** Abbreviations: CDW, cell dry weight; CO_2_, offgas carbon dioxide; pO_2_, dissolved oxygen; CN, cell number; DAC, fraction of damaged cells; CV, cell volume; DHAP, dihydroxyacetone phosphate; G6P, glucose-6-phosphate; GA3P, glyceraldehyde-3-phosphate; HOM, homoserine; ASP, aspartate; VAL, valine.

In a further experiment we cultivated *C. glutamicum* in a long-term batch for 250 h to provoke cell lysis by prolonged glucose limitation (Figure 3). Next to the standard process and analytical data we also analyzed the cell population with regard to cell number and volume as well as fractions of intact and damaged cells by flow cytometric measurements after selective staining with appropriate fluorescence dyes. It can be seen that the percentage of cells with a non-intact membrane is under 1% within 140 h of cultivation. Moreover the absolute cell number increases during that time while the cell dry weight remains approximately constant when finishing exponential growth after 20 h. This difference can be explained by a morphological change of the cells after carbon limitation which is accompanied by a decreasing intracellular volume (cf. Figure 3). The change in morphology can also be observed when *C. glutamicum* cells are cultivated in a microfluidic chip and growth of microcolonies is directly followed by time-lapse microscopy [20]. After 140 h of bioreactor cultivation the cell number rapidly drops, i.e. a very long period of glucose limitation (> 120 h) is necessary to provoke at least some cell lysis.

Most importantly, in none of the executed experiments (cf. Table 1), any kind of energy metabolite, i.e. AMP, ADP, ATP, NAD(H) and NADP(H), was found in the culture medium of all four organisms. Taking additionally the very different metabolite dynamics into account strongly argues against the cell lysis hypothesis.

### Destruction of cell integrity during sample processing

As a second hypothesis, we assume that the culture supernatant is completely free of metabolites throughout the whole cultivation process. Hence, only the combination of environmental changes during sampling (pH, temperature, pO_2_) and physical energy input (centrifugation, filtration) during cell separation can lead to the observed loss of metabolites, which would then originally belong to intracellular pools of viable cells.

**Figure 4:**
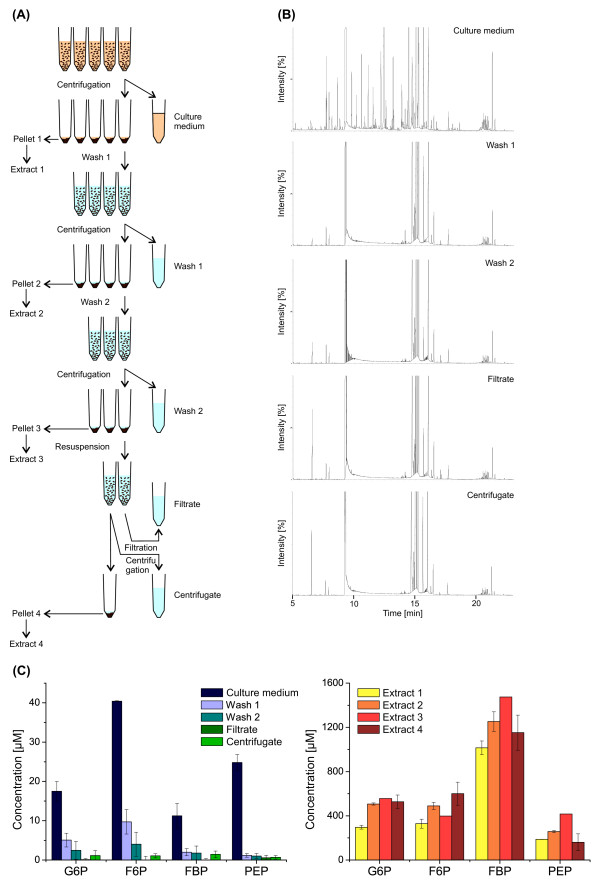
**Impact of sampling procedure on the cell integrity of *E. coli *WT. (A)** Different sample treatments for cell washing and separation. **(B)** Untargeted GC-TOF-MS analysis of resulting cell-free supernatants. Here the mass peaks detected in the washing, filtrate and centrifugate samples are due to derivatized compounds of the washing solution. **(C)** Targeted LC-ESI-MS/MS analysis of resulting cell-free supernatants and cell extracts focusing on specific sugar phosphates from glycolysis. Abbreviations: G6P, glucose-6-phosphate; F6P, fructose-6-phosphate; FBP, fructose-1,6-bisphosphate; PEP, phosphoenolpyruvate.

In order to test this hypothesis, first a culture sample was prepared by repeated washing with fresh 37°C glucose medium to guarantee that no metabolites were present in the extracellular volume (Figure 4A). Then two different cell separation methods, i.e. filtration and centrifugation, were applied to this sample. For all processing steps the resulting cell-free supernatants were analyzed for metabolites by untargeted GC-TOF-MS and targeted LC-ESI-MS/MS (Figure 4B and 4C, left). It can be seen that the extracellular concentration of all metabolites significantly decreased along the washing steps and, more importantly, also after the centrifugation and filtration step no sudden increase in the metabolite concentrations was detectable. Nevertheless, small amounts of sugar phosphates were also found in the washing supernatants. This is most likely due to an ongoing cell metabolism converting the glucose of the washing solution and, comparable to the situation in the bioreactor, excreting metabolites in the surrounding medium.

Now to exclude the possibility that intracellular metabolites may already leave the cells during the washing procedure also the intracellular concentrations were monitored in each resulting cell pellet following extraction and targeted LC-ESI-MS/MS analysis (Figure 4C, right). Here it was found that the respective intracellular concentrations did not drop for any metabolite and hence no metabolite loss occurred during the sample treatment. In fact, the data rather shows slightly increasing intracellular pools along the extracts, which in turn can be explained by the active cell metabolism.

### Metabolite excretion under growth limiting conditions

From the results shown above we can already conclude that:

1) Cell lysis as a potential source for the metabolites in the culture medium cannot be fully excluded, but does not substantially contribute to the high amount of intermediates detected, especially in the exponential growth phase.

2) A reduction of cell integrity during sampling and hence a misinterpretation of the measured data can be excluded.

Hence the majority of intermediates in the culture medium must stem from live and metabolically active cells and is transported via active or passive transport reactions through the cell membrane. For an overview regarding the current knowledge on transport reactions based on the functional genome annotations of all four organisms under investigation see the Additional file 1: Table S2.

Since it goes far beyond this investigation to fully understand the precise mechanistic principle behind this phenomenon we first tried to understand under which metabolic conditions such metabolite transport is provoked.

**Figure 5:**
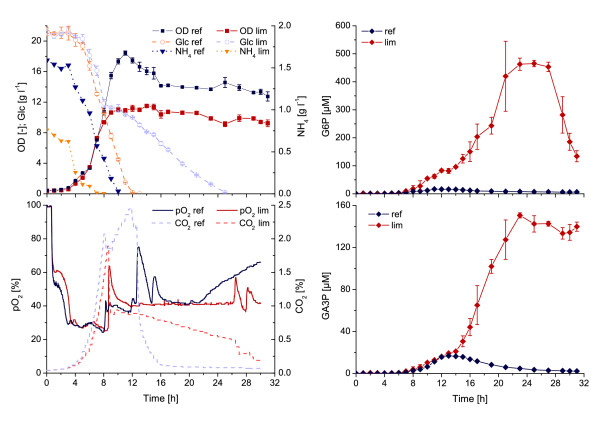
**Impact of limited biomass formation and carbon excess on the exometabolome of *E. coli *WT during batch cultivations on defined media with 20 g l**^**-1**^**glucose.** An ammonia-limited culture (lim) is compared to a reference culture (ref) with 2.5 g l^-1^ and 5 g l^-1^ ammonium sulfate, respectively. Abbreviations: OD, optical density; Glc, glucose; NH_4_, ammonia; pO_2_, dissolved oxygen; CO_2_, offgas carbon dioxide; G6P, glucose-6-phosphate; GA3P, glyceraldehyde-3-phosphate.

In a further experiment, we monitored the exometabolome dynamics during the enhanced conditions of carbon overflow metabolism provoked by limitation of the nitrogen supply following reduced biomass growth and excess of glucose (Figure 5). During ammonia-limited culture, biomass formation stopped immediately after depletion of the ammonia source and glucose uptake decreased, whereas the reference culture showed continuous growth until the glucose had been completely utilized. Again an accumulation of central carbon metabolism intermediates in the culture medium was observed in both experiments, but most surprisingly a great increase in the amount of intermediates was detected in the ammonia-limited culture when biomass growth entered the stationary phase und glucose consumption continued in a linear fashion.

### Extended overflow metabolism

Cultivating cells in bioreactors *per se* enforces high concentration gradients for all intermediates, i.e. high driving forces between the intracellular environment and the highly diluted culture medium. This situation is most pronounced in growth phases with high metabolic activity (exponential growth phase) and under conditions of limited biomass formation with simultaneous excess of substrates.

**Figure 6:**
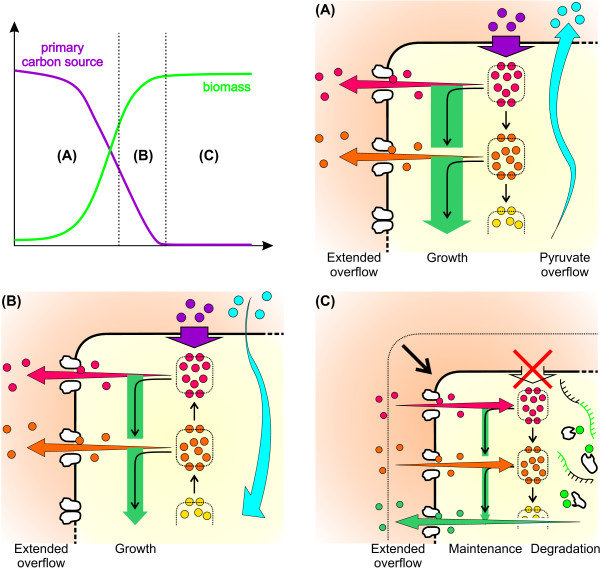
**Extended overflow metabolism during classical batch cultivation. (A)** State under carbon excess of the primary carbon source. Growth is exponential but due to a misbalance between carbon uptake and consumption an overflow of primary by-products (blue circles) and metabolites (red and orange circles) occurs. **(B)** State under limited growth conditions involving re-uptake of primary by-products and ongoing overflow of metabolites. **(C)** State under primary carbon source and by-product limitation. Growth is stationary and accompanied by a decrease in cell size, re-uptake of metabolites, protein and mRNA degradation and overflow of amino acids.

Now, from a mechanistic point of view, it seems that under excess conditions of the primary carbon source (e.g. glucose) and provided that carbon uptake is not inhibited, the cell is directed to metabolize the carbon source at high rates. However, as appropriate carbon conversion might be hampered by maximal enzyme activities, thermodynamical constraints or metabolic regulation, some intermediate pools tend to accumulate. Finally, this results in a kind of “extended” overflow metabolism including primary by-products such as acetate or ethanol but also central metabolic intermediates, which are then transported out of the cell according to a certain active or passive transport mechanism (Figure 6A).

In the subsequent transitory phase, uptake of the primary carbon source is still constant but biomass formation is decreased due to some external growth limiting factor (e.g. ammonia). Additionally, primary by-products are reused, and metabolite overflow continues in a reduced manner (Figure 6B). However, metabolite overflow in this state can also be much higher as shown in the ammonia-limited cultivation experiments (cf. Figure 5).

Finally, when the primary carbon source and by-products are exhausted the cell’s metabolism is reprogrammed to maintenance metabolism. This stationary phase is accompanied by a morphological change towards smaller cells (cf. Figure 3). At this point it can be speculated that degradation of proteins non-essential for the central metabolism, e.g. membrane proteins, occurs to directly supply the necessary amino acids for protein synthesis (Figure 6C). In fact, this kind of starvation response was recently proposed for eukaryotic organisms [21]. This may also explain the accumulation of amino acids such as L-valine in the stationary phase, which then would primarily result from an overflow of amino acids stemming from intracellular protein degradation. Moreover, it is likely that ribosomal RNA is degraded due to a down-regulation of the whole protein synthesis machinery and direct supply of nucleic acid components for repair purposes. Indeed, in nearly all cultivations an increase in the uracil level was found at the beginning of the stationary growth phase (cf. Additional file 1: Figures).

### Impact on intracellular metabolite quantification

One of the major obstacles when aiming at intracellular metabolite quantification is metabolite “leakage”, which describes the effect of metabolite loss during biomass quenching. Noteworthy, leakage is induced as a consequence of the quenching procedure (i.e. sum of factors including temperature, osmotic pressure and organic solvent) leading to a change in cell membrane integrity [22].

In order to circumvent the leakage problem an integrated sampling method was recently proposed [23], which relies on a simultaneous quenching and extraction of a metabolome sample. The idea of whole-broth sampling was later extended by also accounting for extracellular metabolite abundances [14,15]. By this so called “differential method” an intracellular metabolite concentration is simply calculated as the difference of the total broth concentration and the cell-free medium concentration. However, due to the fact that the intracellular volume and the volume of the cultivation media differ by many orders of magnitude this strategy involves the risk that even small extracellular concentrations make it difficult to achieve a reliable quantification of intracellular metabolites. In fact, this is already true for the majority of experimental states during the batch cultivations shown here (cf. Table 1 and Additional file 1: Figures). As a final example:

1) The already introduced example data is considered (cf. section on cell lysis): *c*_GA3P,intra_ = 0.52 μmol g_CDW_^-1^, CDW_max_ = 7.8 g l^-1^.

2) Assuming a minimum sample volume of *V*_S_ = 1 ml for intracellular metabolite quantification.

The resulting molar fractions of GA3P stemming from the cytosol after total broth extraction and already present in the culture medium before extraction, respectively, are calculated as *n*_GA3P,intra_ = 4.06 nmol and *n*_GA3P,extra_ = 15.04 nmol. Hence nearly 80% of the total molar amount of GA3P in the broth extract is represented by the extracellular metabolites as a consequence of metabolite overflow. The example represents a best case scenario when running this protocol under standard batch conditions. In case of the ammonia-limitation experiment we detected a maximum extracellular concentration of *c*_GA3P,extra_ = 150.5 μM (cf. Figure 5), which would result in a ratio of 3:97 between the intracellular and extracellular molar fractions. These considerations simply lead to the opposite argument that was made in the discussion on the impact of cell lysis.

The differential method was established on basis of chemostat cultivations where the primary carbon source is limited, and hence, metabolite overflow should be significantly lower as compared to batch conditions. Indeed the extracellular concentration of GA3P in chemostat experiments with *E. coli* (*μ* = 0.1 h^-1^) was *c*_GA3P,extra_ = 0.15 μmol g_CDW_^-1^[15], but during our standard batch conditions the detected GA3P concentration ranges from 0.46 ≤ *c*_G6P,extra_ ≤ 2.28 μmol g_CDW_^-1^. This holds true for almost all directly comparable intermediates of glycolysis and TCA-cycle as well as halve of the proteinogenic amino acids.

As an alternative approach, leakage can be quantified and, if necessary, corrected by also analyzing the resulting quenching supernatant after the following cell separation step. However, by taking this extended version of the classical quenching approach already three error-prone concentration measurements for each metabolite are necessary to calculate the final intracellular concentration value. Therefore a thoroughly error propagation analysis, including all accessible influencing factors that finally impact the accuracy of the intracellular metabolite concentration data, is strongly recommended when applying such a protocol [Tillack J et al.: Error Propagation Analysis for Quantitative Intracellular Metabolomics, Submitted].

### Impact on ^13^C-metabolic flux analysis

Currently, metabolic flux analysis based on isotopic tracer experiments (^13^C-MFA) is the standard method for quantifying intracellular fluxes [24,25]. The underlying metabolic network model contains the mass balances and atom transitions of all intermediate pools within the system boundaries, e.g. central metabolism, starting from the isotopic tracer substrate, e.g. ^13^C-labeled glucose. In accordance with the general requirement of metabolic stationarity, all experimentally determined fluxes over the system boundary, i.e. rates for substrate uptake, (by-)product formation and biomass synthesis, should be constant and give rise to an entirely closed carbon balance [26]. Now, in the light of our results the most obvious question is the extent to which the observed metabolite overflow impairs this carbon balance.

To assess the impact of extended metabolite overflow on the total carbon balance we took once again a closer look at one *E. coli* dataset of our study (cf. Additional file 1: Figure S1). The carbon balance at the end of the exponential growth phase is closed up to 94%, i.e. comprising 65.0% C-mol for biomass formation (assuming 0.45% w w^-1^ carbon per gram biomass according to [27]), as well as 19.5% C-mol CO_2_, 4.6% C-mol acetate, 4.2% C-mol pyruvate, 0.2% C-mol other organic acids, 0.1% C-mol sugar phosphates, and < 0.1% C-mol amino acids. Hence less than 1% of the carbon from glucose was shifted into the exometabolome (not including the primary by-products acetate and pyruvate). Here it has to be kept in mind that we took a targeted LC-MS/MS approach and hence only central metabolic intermediates were considered.

However, it is more likely that the remaining 6% C-mol for closing the carbon balance are due to systematic errors, *e.g.*, carbon composition of biomass, dry weight measurements, or evaporation effects during cultivation. Consequently, with respect to the formulation of carbon balances, this result rather emphasizes the importance of identifying the major contributors of metabolite balancing errors than demanding for extensive exometabolome analysis.

Nevertheless, the influence of the exometabolome on the analyses of labeling patterns in intermediates of central carbon metabolism could be of significant more importance than the mere carbon balance. Depending on the specific amount and labeling state of the extracellular intermediate pool at the beginning of a ^13^C-tracer experiment, as well as the exchange rate with the intracellular pool during the experiment, the intracellular labeling dynamics can be significantly affected. This could be of special importance for the isotopic non-stationary method that relies on isotopic transient data, and beyond that, also on accurate measurements of intracellular pool sizes [28].

## Conclusions

The presence of significant amounts of intracellular metabolites in the culture medium during well-defined bioreactor cultivations is a general phenomenon and is not restricted to specific species-dependent properties (e.g. Gram-positive versus Gram-negative, prokaryote versus eukaryote). In more detail, our data show a strong correlation of the metabolite concentrations in the culture medium and the metabolic state of the cell population. Particularly with regard to cultivation phases where growth is reduced and a carbon source is still extensively available, higher amounts of intermediates are released by the cells. Vice versa, when the cells enter a state of prolonged C-source limitation some intermediates are taken up as alternative substrates.

The fact that for all organisms under investigation an entire spectrum of intermediates from the central carbon metabolism was found in the culture medium underlines the need to account for this additional exometabolome in every quantitative analysis directed to intracellular metabolite concentrations and/or dynamic labeling patterns for flux quantification.

In turn, this demands for further investigations on the precise metabolite transport mechanism in order to correctly formulate the atom transition models at the systems boundary including possible labeling exchange caused by simultaneous excretion and uptake of central metabolic intermediates.

## Methods

### Strains and media

In order to keep the influence of the cultivation conditions as small as possible and to enable optimal comparability between the different organisms used, a defined mineral medium was developed in each case as follows. The *E. coli* strain used in this study was K12 W3110. The shake flask medium contained per liter of distilled water: 5 g (NH_4_)_2_SO_4_, 11.6 g K_2_HPO_4_, 9.63 g KH_2_PO_4_, 20 g D-glucose, 98 mg MgSO_4_*7H_2_O, 70 mg CaCl_2_*5H_2_O, 50 mg FeSO_4_*7H_2_O, 17.1 mg MnSO_4_*1H_2_O, 15 mg ZnSO_4_*7H_2_O, 3 mg Na_2_MoO_4_*2H_2_O, 2.5 mg CuSO_4_*5H_2_O, 1.7 mg NiCl_2_*6H_2_O, 0.75 mg AlCl_3_*6H_2_O, 0.6 mg CoCl_2_*6H_2_O, 0.5 mg H_3_BO_3_. The medium was adjusted to pH 7.0 with sodium hydroxide. The bioreactor medium additionally contained 1 ml of 10% (v v^-1^) AF 204 (Sigma) per liter.

The *B. licheniformis* strain used in this study was DSM13D102. The shake flask medium contained per liter of distilled water: 15 g (NH_4_)_2_SO_4_, 17.4 g K_2_HPO_4_, 14.45 g KH_2_PO_4_, 16 g D-glucose, 0.49 g MgSO_4_*7H_2_O, 0.7 g CaCl_2_*5H_2_O, 50 mg FeSO_4_*7H_2_O, 57.6 mg MnCl_2_*4H_2_O, 0.67 NiSO_4_*6H_2_O, 0.65 mg Na_2_MoO_4_*2H_2_O, 0.57 mg CoCl_2_*6H_2_O, 0.3 mg CuSO_4_*5H_2_O, 0.25 mgZnCl_2_, 0.006 mg H_3_BO_3_. The medium was adjusted to pH of 7.6 with sodium hydroxide. The bioreactor medium additionally contained 1 ml of 10% (v v^-1^) AF 204 (Sigma) per liter.

The *S. cerevisiae* strain used in this study was CEN.PK 113-7D. The shake flask medium contained per liter of distilled water: 5 g (NH_4_)_2_SO_4_, 5.8 g K_2_HPO_4_, 4.8 g KH_2_PO_4_, 16 g D-glucose, 0.49 g MgSO_4_*7H_2_O, 0.7 g CaCl_2_*5H_2_O, 50 mg FeSO_4_*7H_2_O, 4.5 mg ZnSO_4_*7H_2_O, 1.03 mg MnCl_2_*4H_2_O, 0.3 mg CoCl_2_*6H_2_O, 0.3 mg CuSO_4_*5H_2_O, 0.4 mg Na_2_MoO_4_*2H_2_O, 0.1 mg H_3_BO_3_, 0.01 mg KI, 15 mg Na_2_EDTA, 0.05 mg D-biotin, 1 mg calcium pantothenate, 1 mg thiamine hydrochloride, 1 mg pyridoxine hydrochloride, 1 mg nicotinic acid, 0.2 mg p-aminobenzoic acid, 25 mg myo-inositol. The medium was adjusted to pH of 5 with hydrochloric acid. The bioreactor medium additionally contained 1 ml of 10% (v v^-1^) AF 204 (Sigma) per liter.

The *C. glutamicum* strain used in this study was DM’1800. For cultivation the defined mineral medium CGXII [29] was used, containing per liter of distilled water: 20 g (NH_4_)_2_SO_4_, 1 g K_2_HPO_4_, 1 g KH_2_PO_4_, 5 g urea, 10 g D-glucose, 13.25 mg CaCl_2_*2H_2_O, 0.25 g MgSO_4_*7H_2_O, 1 mg FeSO_4_*7H_2_O, 1 mg MnSO_4_*H_2_O, 0.02 mg NiCl_2_*6H_2_O, 0.313 mg CuSO_4_*5H_2_O, 1 mg ZnSO_4_*7H_2_O. The medium was adjusted to pH of 7.0 with sodium hydroxide. The bioreactor medium additionally contained 3 ml of 10% (v v^-1^) AF 204 (Sigma) and 1 mL of a 0.2 g l^-1^ biotin stock solution per liter added after sterilization.

Cryocultures of all organisms were stored at −80°C in the specific medium containing 20% (v v^-1^) glycerol.

### Cultivation conditions

For precultivation of *E. coli*, *B. licheniformis* and *S. cerevisiae,* two 1 l shake flasks, each with 100 ml medium, were prepared and inoculated with 1 mL of cryoculture. Precultivation of *E. coli* was carried out at 37°C and 180 rpm for 15 h, for *B. licheniformis* at 39°C and 200 rpm for 8 h and for *S. cerevisiae* at 30°C and 180 rpm for 15 h. The precultures were combined and centrifuged (5 min, 4 500 g, 25°C). The supernatant was decanted and the pellet resolved gently in 50 ml of fresh, warm medium (temperature depending on the organism). 10 ml was then used to inoculate a 1.5 l bioreactor (DASGIP AG, Jülich, Germany) with a working volume of 1 l. Bioreactors for the cultivation of *C. glutamicum* were inoculated directly with 2 mL of cryoculture without performing any preculture. All cultivations were carried out in batch mode at constant air flow (1 vvm) and constant temperature (according to the precultivations). The pH value was maintained at the organism-specific optimum (cf. Section 2.2) by adding 4 M NaOH and 4 M HCl, respectively. Aerobic process conditions (pO_2_ > 40% for all strains, except *C. glutamicum* pO_2_ > 30%), were ensured via stirrer speed control (200–1200 rpm). Dissolved oxygen (Visiferm DO 225, Hamilton), pH (405-DPAS-SC-K80/225, Mettler Toledo) and exhaust gas concentrations of carbon dioxide and oxygen (GA4, DASGIP AG, Jülich, Germany) were measured online. Fractions of ^12^C-CO_2_ and ^13^C-CO_2_ in isotopic labeling experiments were measured online using external FT-IR-analytics (GASMET, Ansyco, Germany).

### Sampling procedures

For culture supernatant analysis, a cell suspension volume of 2 ml was taken up into a 5 ml plastic syringe to withdraw the dead volume of the sample port. 3 ml was then taken up into a fresh 5 ml plastic syringe. 1 ml was centrifuged (60 s, 13 000 rpm, Biofuge pico, Haereus) and the supernatant was squeezed through a sterile filter (polyvinylidene fluoride, 0.2 μm pore size). From this filtrate, 250 μl was transferred in 750 μl of −20°C 60% (v/v) methanol for LC-MS/MS analysis, 50 μl was used for glucose analysis, 250 μl for HPLC analysis and 13 μl for GC-TOF-MS analysis. Prior to analysis, the samples were stored at −20°C. For the analysis of intracellular metabolite concentrations, the cells were separated via centrifugation (10 min, 9500 g, -20°C) and the supernatant was decanted. The cell pellet was resolved in 1 ml of −20°C methanol, 1 ml of 4°C TE buffer (10 mM Tris, 1 mM EDTA, pH 7.0) and 2 ml of −20°C chloroform and incubated for 2 h at −20°C in a Labquake shaker (Reax 2, Heidolph) for extraction. The extraction volume was 50 times the intracellular volume in the pellet detected by Coulter counter analysis (CASY® 1 Modell TT equipped with a 45 μm capillary, Roche Diagnostics). Afterwards the mixture was centrifuged again and the upper phase was used for further analysis.

### Process analysis

Optical density (OD_600_) was measured at 600 nm (Shimadzu, PharmaSpec UV 1700) against 0.9% (w/v) NaCl. Glucose was measured using an enzymatic analysis system (EBIO compact, Eppendorf AG Hamburg). Cell count and cell size were monitored offline via a Coulter counter (CASY® 1 Modell TT, Roche Diagnostics). Viability was measured using the LIVE/DEAD® Bac*Light*^*TM*^ Bacterial Viability and Counting Kit in combination with a flow cytometer (FACSAria II, BD Biosciences). To measure the ammonium concentration, a refractometer (RQflex plus, Merck) with the appropriate ammonium kit (116977, Merck) was used. Organic acids were measured by HPLC (1200 Series, Agilent) using an organic acid column (CS chromatography) with an isocratic elution profile at a temperature of 40°C, a flow rate of 0.5 ml min^-1^ and 0.1 M H_2_SO_4_ as the mobile phase.

### LC-ESI-MS/MS analysis

Culture supernatants and extracts were measured by HPLC (X-LC 3000 Series, Jasco) coupled to a mass spectrometer (API 4000, ABSciex) equipped with a TurboIon spray source. For the analysis of intermediates of the central metabolism, a C18 column (synergy hydro, Phenomenex) was used with eluent A (10 mM tributylamine aqueous solution adjusted pH to 4.95 with 15 mM acetic acid) and eluent B (methanol) at a temperature of 60°C. The elution gradient was as follows: 2 min (100% A), 5 min (80% A), 8 min (80% A), 10 min (65%), 14 min (0% A), 15 min (0% A), 15.5 min (100% A) and 17 min (100% A). Amino acids were analyzed by applying an ion exchange column (Luna SCX, Phenomenex) with eluent A (5% acetic acid) and eluent B (15 mM ammonium acetate aqueous solution adjusted pH to 6.0 with 100% acetic acid) at a temperature of 40°C. The elution gradient was as follows: 10 min (85% A), 17 min (0% A), 25 min (0% A) and 29 min (85%). In both cases the flow rate was 0.45 ml min^-1^ and the injection volume 10 μl. For details regarding MS operation see [30,31]. Both methods were used with a ^13^C-labeled internal standard applying the IDMS method [32]. The internal standard was produced by performing a batch cultivation of *E. coli* with uniformly ^13^C-labled glucose as the sole carbon source.

### GC-TOF-MS analysis

Prior to analysis, 13 μl aliquots of the samples were lyophilized overnight in a Christ LT-105 freeze drier and then stored at −20°C. The dried samples were consecutively derivatized with 50 μl MeOX (20 mg ml^-1^ O-methylhydroxylamine in pyridine) for 90 min at 30°C and 600 rpm in an Eppendorf Thermomixer followed by incubation with an additional 80 μl of MSTFA (N-acetyl-N-(trimethylsilyl)-trifuoroacetamide) for 90 min at 40°C and 600 rpm. For the determination of derivatized metabolites, an Agilent 6890 N gas chromatograph was used coupled to a Waters Micromass GCT Premier high-resolution time-of-flight mass spectrometer. The system was controlled by Waters MassLynx 4.1 software. Injection was performed by a Gerstel MPS 2 controlled by Maestro software. 1 μl sample was injected into a split/splitless injector at 280°C at varying split modes. The GC was equipped with a 30 m Varian FactorFour VF-5 ms + 10 m guard column. Constant Helium flow was set to 1 ml min^-1^. The GC temperature program started at 60°C with a hold time of 2 min, followed by a temperature ramp of +12°C min^-1^ up to the final temperature of 300°C, with a hold time of 8 min. The total run time was therefore 30 min. The transfer line temperature was set to 300°C. The ToF MS was operated in positive electron impact [EI]^+^ mode at an electron energy of 70 eV. The source temperature was set to 180°C. The MS was tuned and calibrated with the mass fragmentation pattern of Heptacosa (heptacosafluoro-tributylamine). During analysis, the accurate masses were corrected to a single point lock mass of CPFB (chloropentafluorobenzene) as an external reference at 201.9609 m/z. Data acquisition was done in centroid mode at a scan rate of 0.09 s and an interscan delay of 0.01, i.e. 10 scans s^-1^. For the identification of known metabolites a baseline noise subtracted fragment pattern was used and compared to the in-house database JuPoD, the commercial database NIST and the freely available database GMD. Unknown peaks were identified by a structural combination of elemental compositions and verified by virtual derivatization and fragmentation of the predicted structure.

## Competing interests

The authors declare that they have no competing interests.

## Authors’ contributions

NP, AN and TL conducted the experiments and analyzed the data; JG performed GC-TOF-MS analysis; SN wrote the paper with substantial input from NP and WW. All authors read and approved the final manuscript.

## Supplementary Material

Additional file 1 Supplementary Information. Click here for file
