# Supplementary material for: Extensive exometabolome analysis reveals extended overflow metabolism in various microorganisms
Source: Microb Cell Fact. 2012 Sep 11;11:122. doi: 10.1186/1475-2859-11-122 (PMC3526501; doi:10.1186/1475-2859-11-122)
Supplement: Additional file 1 — Supplementary Information. [file 1475-2859-11-122-S1.docx]

**Supplementary Information**

**Extensive Exometabolome Analysis Reveals Extended Overflow Metabolism in various Microorganism**

Nicole Paczia^1^, Anke Nilgen^1^, Tobias Lehmann^1^, Jochem Gätgens^1^, Wolfgang Wiechert^1^, Stephan Noack*^1^

Content

[1. Abbreviations 2](#_Toc333312659)

[2. Organism-wide exometabolome analysis 3](#_Toc333312660)

[3. Hypotheses on metabolite occurrence 7](#_Toc333312661)

[4. Metabolite transport 8](#_Toc333312662)

[5. References 9](#_Toc333312663)

Abbreviations

| **Central metabolic intermediates** | |  | **Amino acids** | |
| --- | --- | --- | --- | --- |
| G6P | Glucose-6-phosphate |  | ALA | Alanine |
| F6P | Fructose-6-phosphate |  | LEU | Leucine |
| FBP | Fructose-1,6-bisphosphate |  | VAL | Valine |
| DHAP | Dihydroxyacetone phosphate |  | ASP | Aspartate |
| GA3P | Glyceraldehyde-3-phosphate |  | HOM | Homoserine |
| 13PG | 1,3-phosphoglycerate |  | THR | Threonine |
| 2PG | 2-phosphoglycerate |  | MET | Methionine |
| 3PG | 3-phosphoglycerate |  | LYS | Lysine |
| PEP | Phosphoenolpyruvate |  | ILEU | Isoleucine |
| PYR | Pyruvate |  | MET | Methionine |
| R5P | Ribose-5-phosphate |  | TRP | Tryptophane |
| RU5P | Ribulose-5-phosphate |  | TYR | Tyrosine |
| X5P | Xylulose-5-phosphate |  | PHE | Phenylalanine |
| E4P | Erythrose-4-phosphate |  | GLU | Glutamate |
| S7P | Sedoheptulose-7-phosphate |  | GLN | Glutamine |
| CIT | Citrate |  | PRO | Proline |
| CAN | Cis-aconitate |  | SER | Serine |
| ICIT | Isocitrate |  | GLY | Glycine |
| AKG | α-Ketoglutarate |  | ARG | Arginine |
| SUC | Succinate |  | HIS | Histidine |
| FUM | Fumarate |  |  |  |
|  |  |  |  |  |
| **Others** | |  |  |  |
| ACT | Acetate |  |  |  |
| ETH | Ethanol |  |  |  |
| ORO | Orotate |  |  |  |
| URA | Uracil |  |  |  |

Organism-wide exometabolome analysis


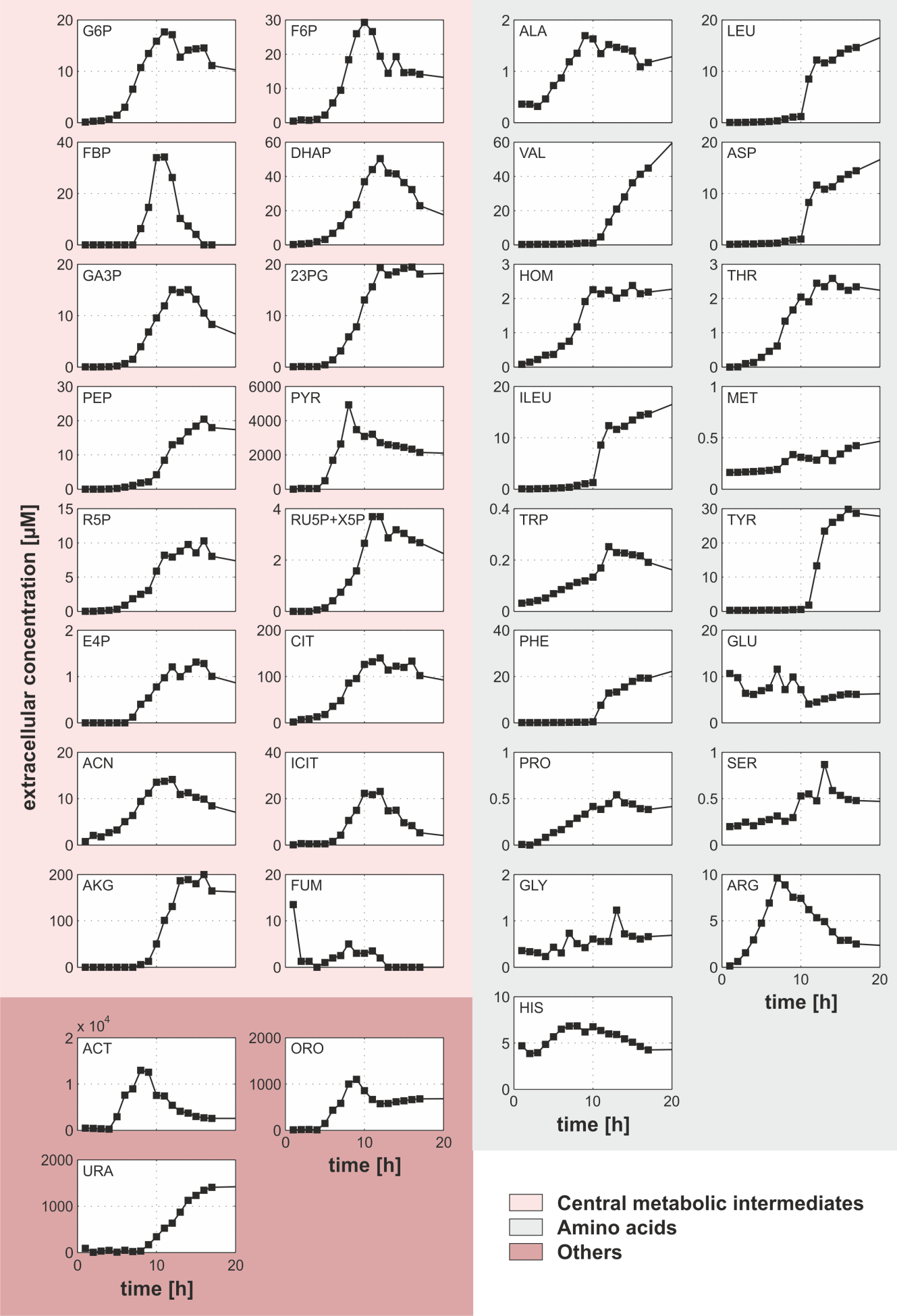


**Figure S1:** Exometabolome analysis of intermediates and free amino acids from central metabolism during batch-cultivation of *E. coli* WT on defined media with 20 g l^-1^ glucose.

**
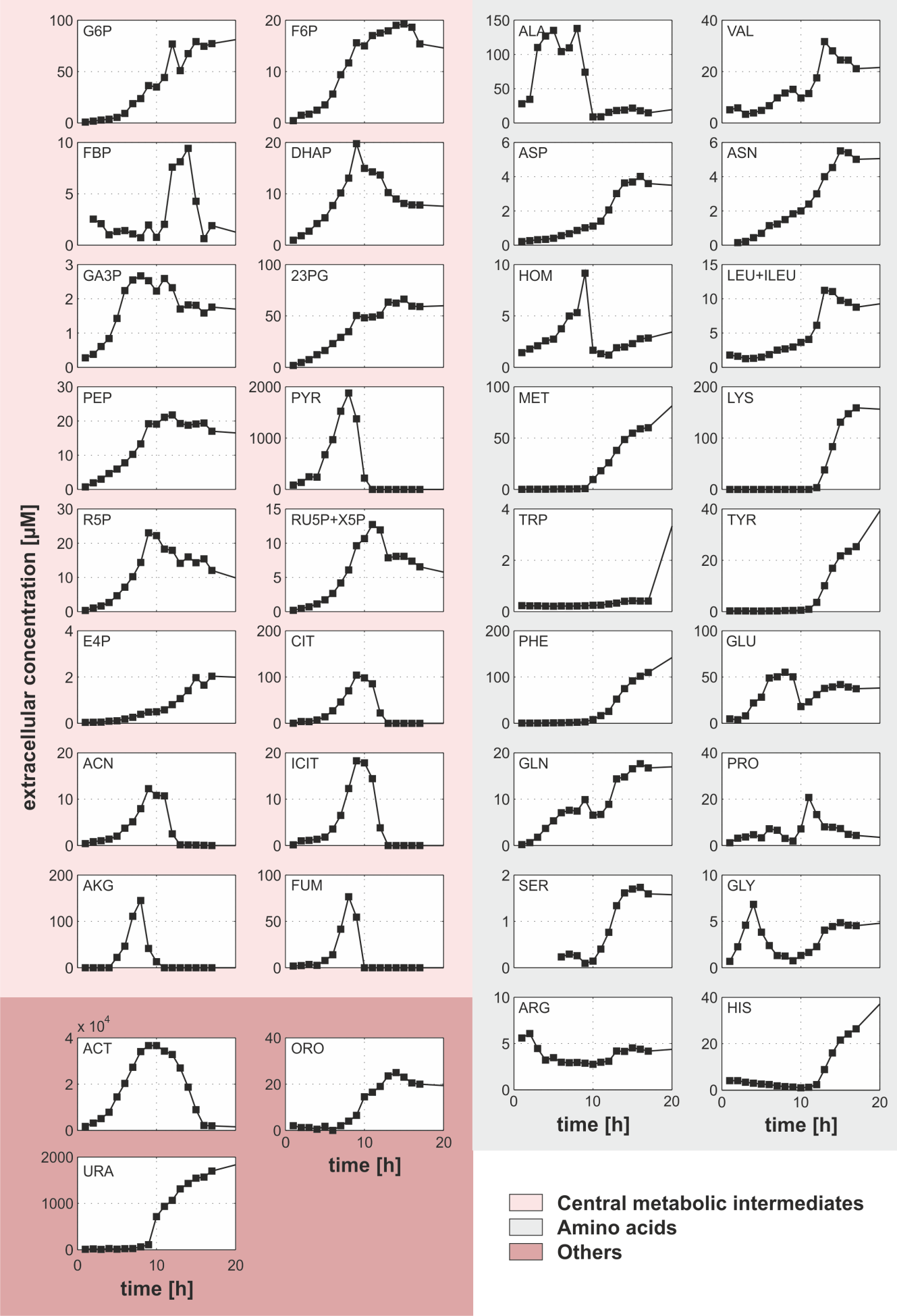
**

**Figure S2:** Exometabolome analysis of intermediates and free amino acids from central metabolism during batch-cultivation of *B. licheniformis* WT on defined media with 16 g l^-1^ glucose.

**
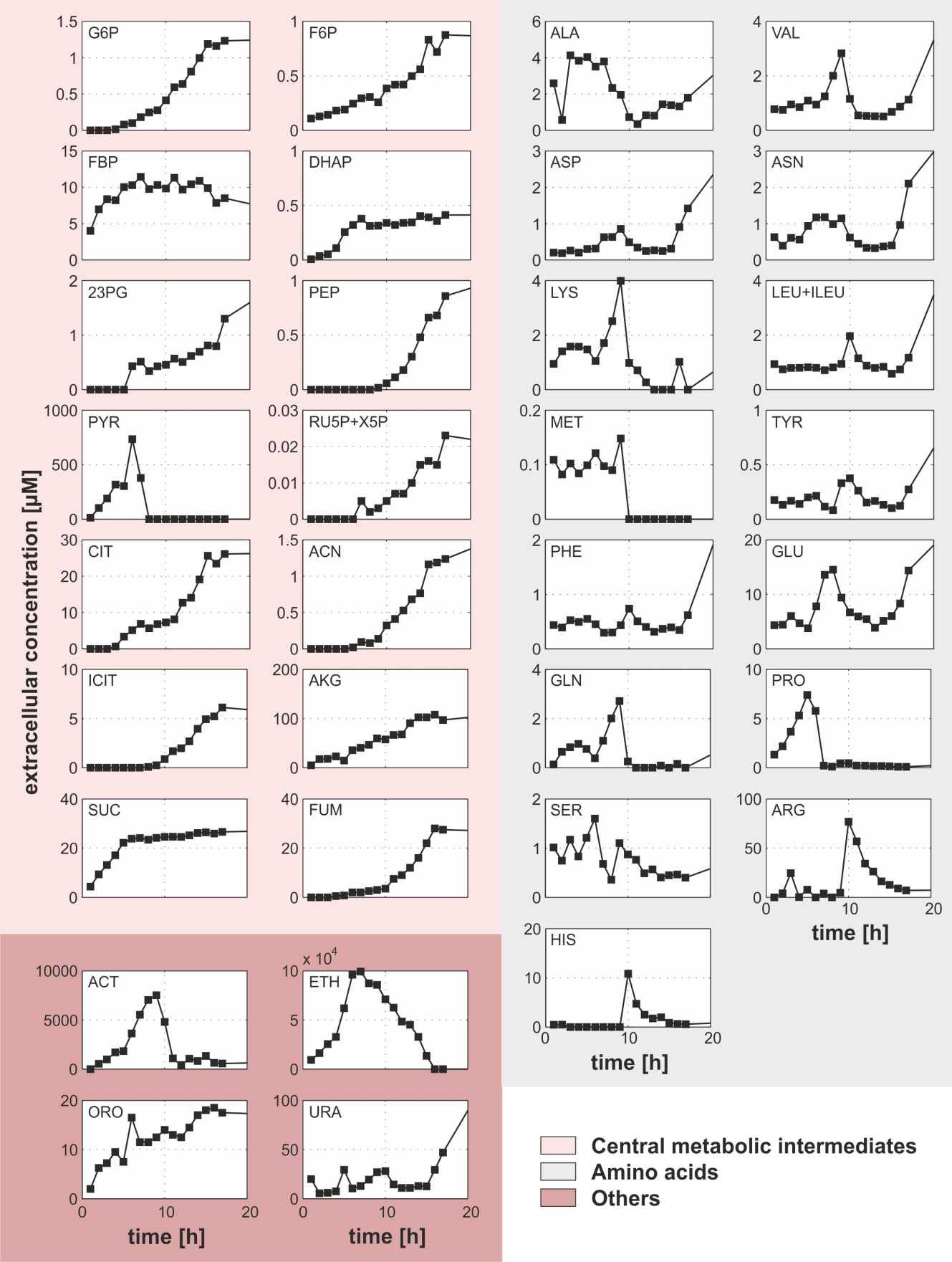
**

**Figure S3:** Exometabolome analysis of intermediates and free amino acids from central metabolism during batch-cultivation of *S. cerevisiae* WT on defined media with 16 g l^-1^ glucose.

**
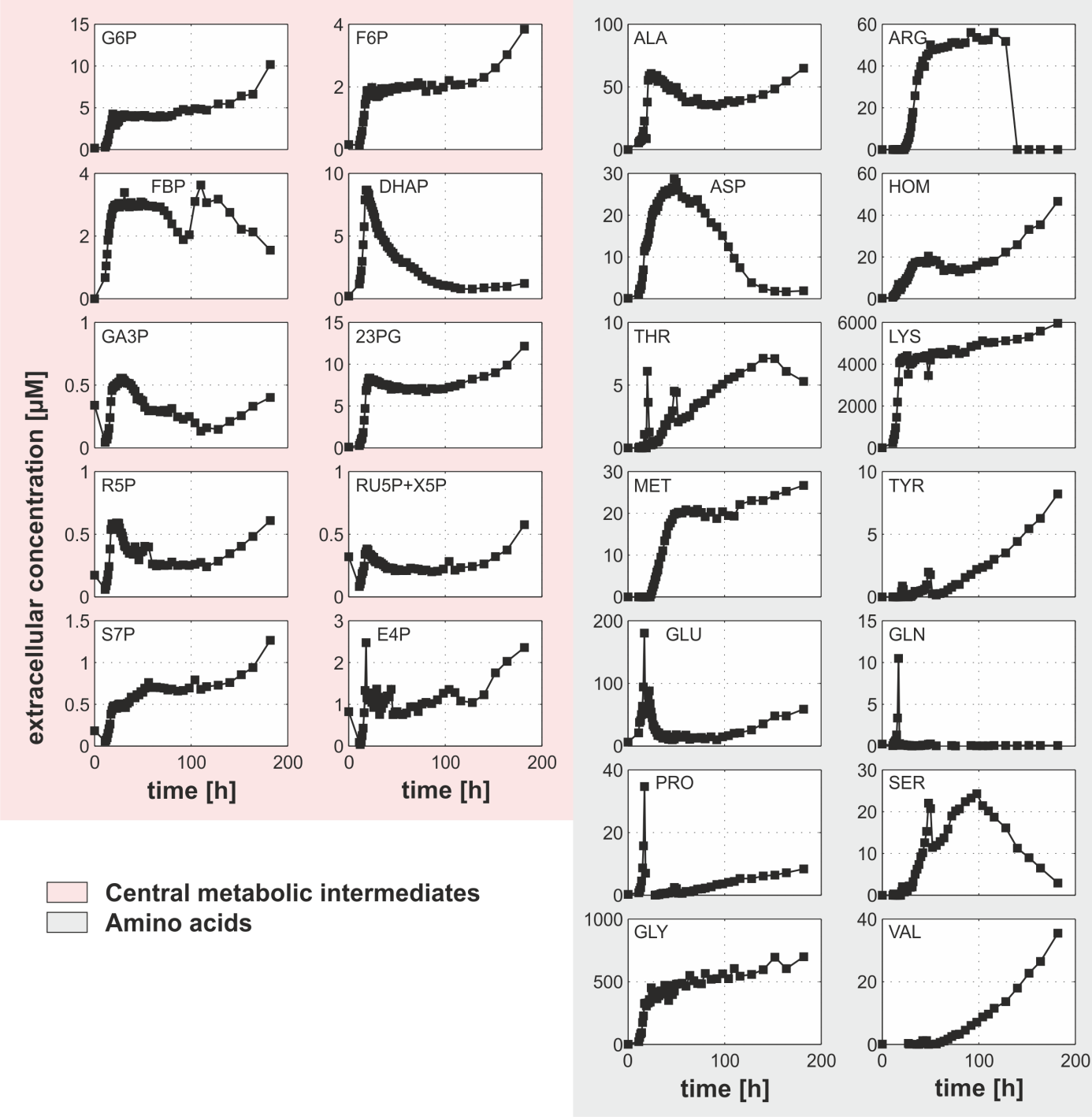
**

**Figure S4:** Exometabolome analysis of intermediates and free amino acids from central metabolism during batch-cultivation of *C. glutamicum* DM1800 on defined media with 10 g l^-1^ glucose.

Hypotheses on metabolite occurrence

**Table S1:** Theoretically lysed biomass to explain the occurrence of extracellular metabolites in the culture broth during *E. coli* WT batch cultivations. Applying mass balancing the measured concentration data of intracellular metabolites found in the culture medium were set into relation with the specific minimum and maximum intracellular concentration value reported for *E. coli* WT so far (shown in bold blue and red, respectively). If necessary reference values were recalculated to biomass specific values using the reported specific intracellular volumes (see table footer). The maximal biomass dry weight in our experiment was CDW_max_ ≈ 8 g.

| **Central**  **metabolic**  **intermediate** | **Measured**  **Culture**  **medium**  **concentration^a^**  [µmol l^-1^] | **Reference intracellular concentration**  [µmol g_CDW_^-1^] | | | | | | | **Theoretically**  **lysed biomass^g^**  [g_CDW_] | |
| --- | --- | --- | --- | --- | --- | --- | --- | --- | --- | --- |
|  |  | [[1](#_ENREF_1)]^b^ | [[2](#_ENREF_2)]^c^ | [[3](#_ENREF_3)]^d^ | [[4](#_ENREF_4)]^e^ | [[5](#_ENREF_5)] | [[6](#_ENREF_6)] | [[7](#_ENREF_7)]^f^ | min | max |
| G6P | 17.65 ± 0.53 | 0.61 | **8.28** | **0.47** | - | 1.42 | 1.22 | - | **2.1** | **37.6** |
| F6P | 29.29 ± 0.60 | 0.70 | **1.43** | **0.21** | - | 0.38 | 0.27 | - | **20.5** | **139.5** |
| FBP | 34.21 ± 0.72 | 9.15 | 0.65 | 0.65 | **37.50** | - | 0.67 | **0.43** | **0.9** | **79.6** |
| GA3P | 15.04 ± 0.17 | - | **0.52** | **0.52** | **0.12** | 0.17 | - | - | **28.9** | **125.3** |
| 2/3PG | 19.45 ± 1.55 | - | - | - | **3.75** | 1.65 | **1.24** | - | **5.2** | **15.7** |
| PEP | 20.43 ± 0.28 | - | **6.36** | 0.94 | 0.45 | 1.61 | 1.22 | **0.08** | **3.2** | **255.4** |
| AKG | 2.27 ± 0.35 | - | - | - | **1.10** | **0.31** | - | - | **2.1** | **7.3** |
| FUM | 12.45 ± 2.31 | - | - | - | **0.30** | **0.22** | - | - | **41.5** | **56.6** |

^a^The time dependent maximum is taken from Table 1.

^b^2.78 ml g_CDW_^-1^

^c^2.38 ml g_CDW_^-1^

^d^2.6 ml g_CDW_^-1^

^e^2.5 ml g_CDW_^-1^

^f^2.15 ml g_CDW_^-1^

^g^The working volume of the bioreactor was 1 l.

Metabolite transport

**Table S2:** Comparison between intermediate pools measured extracellular during batch cultivation in the culture medium and current knowledge on corresponding transport reactions. Red areas indicate mismatches between intermediates measured in this study and annotations in organism specific genome scale models.

|  | ***E. coli***  **(K12 W3110)** | | ***C. glutamicum***  **(DM1800)** | | ***B. licheniformis***  **(DSM13D102)** | | ***S. cerevisiae***  **(CEN.PK 113-7D)** | |
| --- | --- | --- | --- | --- | --- | --- | --- | --- |
| **Central**  **metabolic**  **intermediates** | Measured  extracellular  (this study) | Annotated  transport  reaction [[8](#_ENREF_8)] | Measured  extracellular  (this study) | Annotated  transport  reaction [[9](#_ENREF_9)] | Measured  extracellular  (this study) | Annotated  transport  reaction [[10](#_ENREF_10)]^c^ | Measured  extracellular  (this study) | Annotated  transport  reaction [[11](#_ENREF_11)] |
| G6P | + | + | + |  | + | + | + |  |
| F6P | + | + | + |  | + | + | + |  |
| FBP | + |  | + |  | + |  | + |  |
| GA3P | + |  | + |  | + |  |  |  |
| DHAP | + |  | + |  | + |  | + |  |
| 13PG | +^a^ |  | +^a^ |  | +^a^ |  | +^a^ |  |
| 3PG |  |  |  |  |  | + |  |  |
| 2PG |  |  |  |  |  | + |  |  |
| PEP | + |  |  |  |  | + | + |  |
| PYR | + | + |  |  | + | + | + | + |
| R5P | + | + | + |  | + |  |  |  |
| RU5P | +^a^ |  | +^a^ |  | +^a^ |  | +^a^ |  |
| X5P |  |  |  |  |  |  |  |  |
| E4P | + |  | + |  | + |  |  |  |
| S7P |  |  | + |  |  |  |  |  |
| CIT | + | + |  | + | + | + | + | + |
| CAN | + |  |  |  | + |  | + |  |
| ICIT | + |  |  |  | + |  | + |  |
| AKG | + | + |  |  | + | + | + | + |
| SUC |  | + |  |  |  | + | + | + |
| FUM | + | + |  |  | + | + | + | + |
|  |  |  |  |  |  |  |  |  |
| **Amino acids** |  |  |  |  |  |  |  |  |
| SER | + | + | + | + | + | + | + | + |
| GLY | + | + | + |  | + | + |  | + |
| ALA | + | + | + | + | + | + | + | + |
| VAL | + | + | + | + | + | + | + | + |
| LEU | +^a^ | + |  | + | +^a^ | + | +^a^ | + |
| ILEU |  | + |  | + |  | + |  | + |
| ASP | + | + |  | + | + | + | + | + |
| ASN |  | + | + |  | + | + | + | + |
| HOM | + | + | + |  | + |  |  |  |
| THR | + | + | + | + |  | + |  | + |
| MET | + | + | + | + | + | + | + | + |
| LYS |  | + | +^b^ | + | + | + | + | + |
| TRP | + | + |  | + | + | + |  | + |
| PHE | + | + |  |  | + |  | + | + |
| TYR | + | + | + | + | + | + | + | + |
| GLU | + | + | + | + | + | + | + | + |
| GLN |  | + | + | + | + | + | + | + |
| PRO | + | + | + | + | + | + | + | + |
| ARG | + | + | + | + | + | + | + | + |
| HIS | + | + |  |  | + | + | + | + |

^a^ intermediates are not separable by the applied analytics

^b^ L-lysine production strain

^c^ annotation based on the published genome scale model for *B. subtilis*

**… Table S2:** Continued.

|  | ***E. coli***  **(K12 W3110)** | | ***C. glutamicum***  **(DM1800)** | | ***B. licheniformis***  **(DSM13D102)** | | ***S. cerevisiae***  **(CEN.PK 113-7D)** | |
| --- | --- | --- | --- | --- | --- | --- | --- | --- |
| **Others** | Measured  extracellular  (this study) | Annotated  transport  reaction [[8](#_ENREF_8)] | Measured  extracellular  (this study) | Annotated  transport  reaction [[9](#_ENREF_9)] | Measured  extracellular  (this study) | Annotated  transport  reaction [[10](#_ENREF_10)]^c^ | Measured  extracellular  (this study) | Annotated  transport  reaction [[11](#_ENREF_11)] |
| ACT | + | + |  | + | + | + | + | + |
| ETH |  | + |  |  |  | + | + | + |
| ORO | + | + |  |  | + |  | + |  |
| URA | + | + |  |  | + | + | + | + |

^a^ Intermediates are not separable by the applied analytics

^b^ L-lysine production strain

^c^ Annotation based on the published genome scale model for *B. subtilis*

References

1. Buchholz A, Takors R, Wandrey C: **Quantification of intracellular metabolites in Escherichia coli K12 using liquid chromatographic-electrospray ionization tandem mass spectrometric techniques.** *Analytical biochemistry* 2001, **295:**129-137.

2. Chassagnole C, Noisommit-Rizzi N, Schmid JW, Mauch K, Reuss M: **Dynamic modeling of the central carbon metabolism of Escherichia coli.** *Biotechnology and Bioengineering* 2002, **79:**53-73.

3. Hiller J, Franco-Lara E, Weuster-Botz D: **Metabolic profiling of Escherichia coli cultivations: evaluation of extraction and metabolite analysis procedures.** *Biotechnology letters* 2007, **29:**1169-1178.

4. Bennett BD, Kimball EH, Gao M, Osterhout R, Van Dien SJ, Rabinowitz JD: **Absolute metabolite concentrations and implied enzyme active site occupancy in Escherichia coli.** *Nature chemical biology* 2009, **5:**593-599.

5. Heijnen JJ: **Impact of thermodynamic principles in systems biology.** *Adv Biochem Eng Biotechnol* 2010, **121:**139-162.

6. De Mey M, Taymaz-Nikerel H, Baart G, Waegeman H, Maertens J, Heijnen JJ, van Gulik WM: **Catching prompt metabolite dynamics in Escherichia coli with the BioScope at oxygen rich conditions.** *Metabolic Engineering* 2010, **12:**477-487.

7. Park C, Lee Y, Lee SY, Oh HB, Lee J: **Determination of the Intracellular Concentrations of Metabolites in Escherichia coli Collected during the Exponential and Stationary Growth Phases using Liquid Chromatography-Mass Spectrometry.** *Bulletin of the Korean Chemical Society* 2011, **32:**524-530.

8. Orth JD, Conrad TM, Na J, Lerman JA, Nam H, Feist AM, Palsson B: **A comprehensive genome-scale reconstruction of Escherichia coli metabolism--2011.** *Mol Syst Biol* 2011, **7:**535.

9. Shinfuku Y, Sorpitiporn N, Sono M, Furusawa C, Hirasawa T, Shimizu H: **Development and experimental verification of a genome-scale metabolic model for Corynebacterium glutamicum.** *Microb Cell Fact* 2009, **8:**43.

10. Henry CS, Zinner JF, Cohoon MP, Stevens RL: **iBsu1103: a new genome-scale metabolic model of Bacillus subtilis based on SEED annotations.** *Genome Biol* 2009, **10:**R69.

11. Mo ML, Palsson BO, Herrgård MJ: **Connecting extracellular metabolomic measurements to intracellular flux states in yeast.** *BMC Syst Biol* 2009, **3:**37.
